# Evaluating the Gaps in Nutritional Care for Cancer Patients: Insights from the CIPOMO (Collegio Italiano Primari Oncologi Medici Ospedalieri) National Survey

**DOI:** 10.3390/nu18162593

**Published:** 2026-08-07

**Authors:** Federica Grosso, Giuseppe Aprile, Sandro Barni, Sergio Bracarda, Alessandro Bertolini, Gianfranco Filippelli, Carlo Garufi, Silvana Leo, Alberto Scanni, Maria Giuseppa Sarobba, Rosa Rita Silva, Luisa Fioretto, Paolo Tralongo

**Affiliations:** 1 Mesothelioma, Melanoma and Rare Cancer Unit, IRCCS AOU SS Antonio e Biagio e Cesare Arrigo, 15121 Alessandria, Italy; 2 Department of Oncology, Ospedale Universitario S. Maria della Misericordia, Azienda Sanitaria Universitaria Friuli Centrale (ASUFC), 33100 Udine, Italy; 3 Medical Oncology Unit, Ospedale Treviglio, ASST Bergamo Ovest, 24047 Treviglio, BG, Italy; 4 Ospedale Santa Maria, 05100 Terni, Italy; 5 Institute for Social Security (ISS), 47893 Cailungo, San Marino; 6 Medical Oncology Unit, Ospedale di Paola, ASP Cosenza, 87027 Paola, Italy; 7 Medical Oncology Unit, Azienda Ospedaliera San Camillo Forlanini, 00152 Rome, Italy; 8 Medical Oncology Unit, Ospedale Vito Fazzi, 73100 Lecce, Italy; 9 Medical Oncology Unit, Ospedale Fatebenefratelli-Oftalmico, 20121 Milan, Italy; 10 Oncology Unit, Ospedale San Francesco, ATS Sardinia, 08100 Nuoro, Italy; 11 Medical Oncology Unit, Ospedale di Fabriano, AST Ancona, 60044 Fabriano, Italy; 12 Medical Oncology Unit, Oncology Department, AUSL Toscana Centro, 50139 Florence, Italy; 13 Medical Oncology Unit, Oncology Care Network, Azienda Sanitaria Provinciale 8, 96100 Syracuse, Italy

**Keywords:** oncology, malnutrition, nutritional assessment, dedicated nutritional pathways, multidisciplinary care

## Abstract

Malnutrition, a key factor in oncology, affects 20–70% of cancer patients and influences both cancer risk and outcomes, being linked to decreased treatment tolerance, higher toxicity, lower quality of life, and shorter survival. Although international guidelines recommend early and systematic nutritional screening, its adoption in clinical practice remains inconsistent. This study, promoted by the Italian College of Hospital Medical Oncology Directors (CIPOMO), aimed to provide a national snapshot of current organizational practices regarding nutritional screening, assessment, and management in Italian oncology centers. A 15-item survey was sent to 165 centers to investigate organizational practices and awareness of nutrition and physical activity in cancer care. Data were collected and analyzed as overall percentages. A total of 100 centers responded (61%). Although a nutritional assessment is routinely conducted at diagnosis in 67% of centers (*n* = 67), only 31 centers perform it for all patients. The remaining centers limit nutritional assessment to high-risk or selected patient groups. Moreover, although 86% of centers have dedicated professionals, multidisciplinary integration remains limited. Validated nutritional screening tools are used in 69% of centers, but follow-up reassessment is rarely systematic (15%). However, awareness of the importance of nutrition is high, with nearly all centers supporting educational interventions and dedicated nutritional pathways. Despite this high level of awareness, a significant gap remains between guideline recommendations and daily clinical practice. Standardizing early nutritional screening, enhancing multidisciplinary collaboration, and ensuring equitable and consistent access to nutritional services are crucial to fully integrating nutrition into oncology care and improving patient outcomes.

## 1. Introduction

Nutrition plays a central role in oncology and is increasingly recognized as an essential component of comprehensive, patient-centered cancer care. Beyond preventing and treating malnutrition, adequate nutritional support may improve treatment tolerance and adherence, preserve functional status and quality of life, and ultimately contribute to better clinical outcomes and survival throughout the disease trajectory [1,2,3]. In addition, growing evidence suggests that diet and nutritional status may influence both cancer risk and prognosis [4,5,6,7].

Cancer patients are particularly vulnerable to nutritional impairment. The prevalence of malnutrition ranges from 20% to over 70%, depending on tumor type and location [8], and is associated with adverse outcomes including increased treatment toxicity, reduced tolerance to anticancer therapies, poorer quality of life, and shorter survival [9,10]. It is estimated that malnutrition directly contributes to death in 10–20% of cancer patients [8]. Manifestations of nutritional impairment are highly prevalent: weight loss affects approximately 65% of patients, with losses ranging from 1 to 10 kg in the six months preceding diagnosis [11]; anorexia occurs in around 40% of cases and is more common in advanced disease [11]; cachexia affects 50–80% of patients [12]; and sarcopenia is observed in 20–60%, depending on tumor type [13,14].

Among these conditions, sarcopenia has emerged as a particularly important prognostic factor. A meta-analysis including 1700 patients with solid tumors demonstrated significantly shorter overall survival and progression-free survival among sarcopenic patients [14]. These findings were confirmed by a large observational study involving more than 13,000 cancer patients, which reported a higher and earlier mortality risk in individuals with sarcopenia [15]. Overall, alterations in body composition should not be regarded merely as consequences of advanced disease but as clinically relevant factors that may significantly influence treatment outcomes and survival.

The clinical impact of nutritional impairment extends beyond prognosis. Malnutrition, sarcopenia, and cachexia have been associated with increased treatment-related toxicity, more frequent dose reductions, treatment delays, and premature discontinuation of systemic therapies, potentially compromising treatment efficacy. Furthermore, nutritional deterioration contributes to fatigue, functional decline, and loss of independence, negatively affecting daily activities and psychosocial well-being [10,11,16].

The relationship between cancer and nutritional status is complex and bidirectional. Tumor-related metabolic alterations and systemic inflammation promote catabolism, reduced food intake, and progressive loss of skeletal muscle mass, while worsening nutritional status may further aggravate inflammation, impair immune competence, and reduce the body’s capacity to respond effectively to anticancer treatments. This interaction creates a vicious cycle that may accelerate clinical deterioration if not promptly recognized and addressed [17].

Recent attention has focused on nutritional risk as a dynamic condition that may evolve throughout the disease course. Patients who are nutritionally stable at diagnosis may experience substantial deterioration during treatment due to therapy-related toxicities, disease progression, or declining functional status. Consequently, nutritional assessment should be integrated as a continuous component of cancer care, with repeated nutritional screening and timely interventions throughout the continuum of care [18].

In light of these findings, European and Italian guidelines recommend nutritional screening for all cancer patients at diagnosis, followed, when indicated by risk status, by a comprehensive nutritional assessment and personalized multimodal interventions tailored to the patient’s clinical condition [16,19]. Early implementation of nutritional support, before severe metabolic impairment and functional decline become clinically evident, has been associated with improved quality of life and clinical outcomes [16].

Despite growing evidence and the availability of national and international recommendations, the implementation of structured nutritional pathways in routine oncology practice remains inconsistent, and many patients do not receive systematic nutritional screening or assessment at diagnosis. Several barriers have been identified, including limited nutritional training among oncologists, lack of standardized screening procedures, organizational constraints, and insufficient integration between oncology and clinical nutrition services [16,20,21].

Despite the availability of national and international recommendations, comprehensive information on how nutritional care is organized and implemented across Italian oncology centers remains limited. The present national survey, promoted by the Collegio Italiano dei Primari Oncologi Medici Ospedalieri (CIPOMO), was designed to provide a national snapshot of current organizational practices regarding nutritional screening, assessment, and management in Italian oncology centers. In addition, the survey explored oncologists’ awareness of nutritional care and described the organizational context in which nutritional pathways are implemented, highlighting areas of variability and potential opportunities for improvement.

## 2. Methods

In light of the available scientific recommendations, the CIPOMO working group developed a 15-item multiple-choice questionnaire drawing on current international recommendations and expert clinical experience in nutritional care for oncology patients. The questionnaire explored organizational aspects of nutritional care in routine oncology practice. Because the survey was designed as an exploratory assessment of current practice, no formal questionnaire validation process (including pilot testing, content validity assessment, or test–retest reliability evaluation) was performed prior to its administration.

Eleven questions were used to evaluate current practice across participating centers, and four questions aimed to gather the opinions of participating centers on the importance of nutrition and physical activity for cancer patients (Table 1). The question on physical activity was included as an exploratory item to assess clinicians’ awareness of its role in cancer care. The survey was not designed to collect detailed information on the organization or characteristics of physical activity programs.

The questions required a yes/no answer and, in some cases, additional investigation through multiple-choice questions.

The complete questionnaire is provided in Table 1. The term “validated nutritional screening tool” referred to screening instruments recommended by the ESPEN guidelines, which were explicitly listed in the questionnaire. Other organizational terms (e.g., “structured nutritional care pathway” and “dedicated specialists”) reflected the terminology commonly used in the Italian oncology setting.

A total of 165 centers were invited to participate. The formal invitation was sent by email to the Head of each Oncology Unit affiliated with CIPOMO. The survey was administered electronically using SurveyMonkey. It was distributed on 5 December 2024 and remained open until 8 March 2025, corresponding to a data collection period of approximately three months. During this period, four reminder emails were sent to non-responding centers (on 12 December 2024, 9 January 2025, 17 January 2025, and 17 February 2025) to encourage participation.

Responses were collected in a non-anonymous manner and subsequently analyzed in aggregate. Given the exploratory and descriptive nature of the survey, data were analyzed using descriptive statistics only. No regional subgroup analyses were performed due to the exploratory nature of the survey and because the number of participating centers within individual geographic areas was insufficient to support reliable comparisons.

Categorical variables are presented as frequencies and percentages. No inferential statistical analyses were planned, as the primary objective was to provide a nationwide overview of current organizational practices rather than to test predefined hypotheses or compare subgroups.

## 3. Results

**Figure 1:**
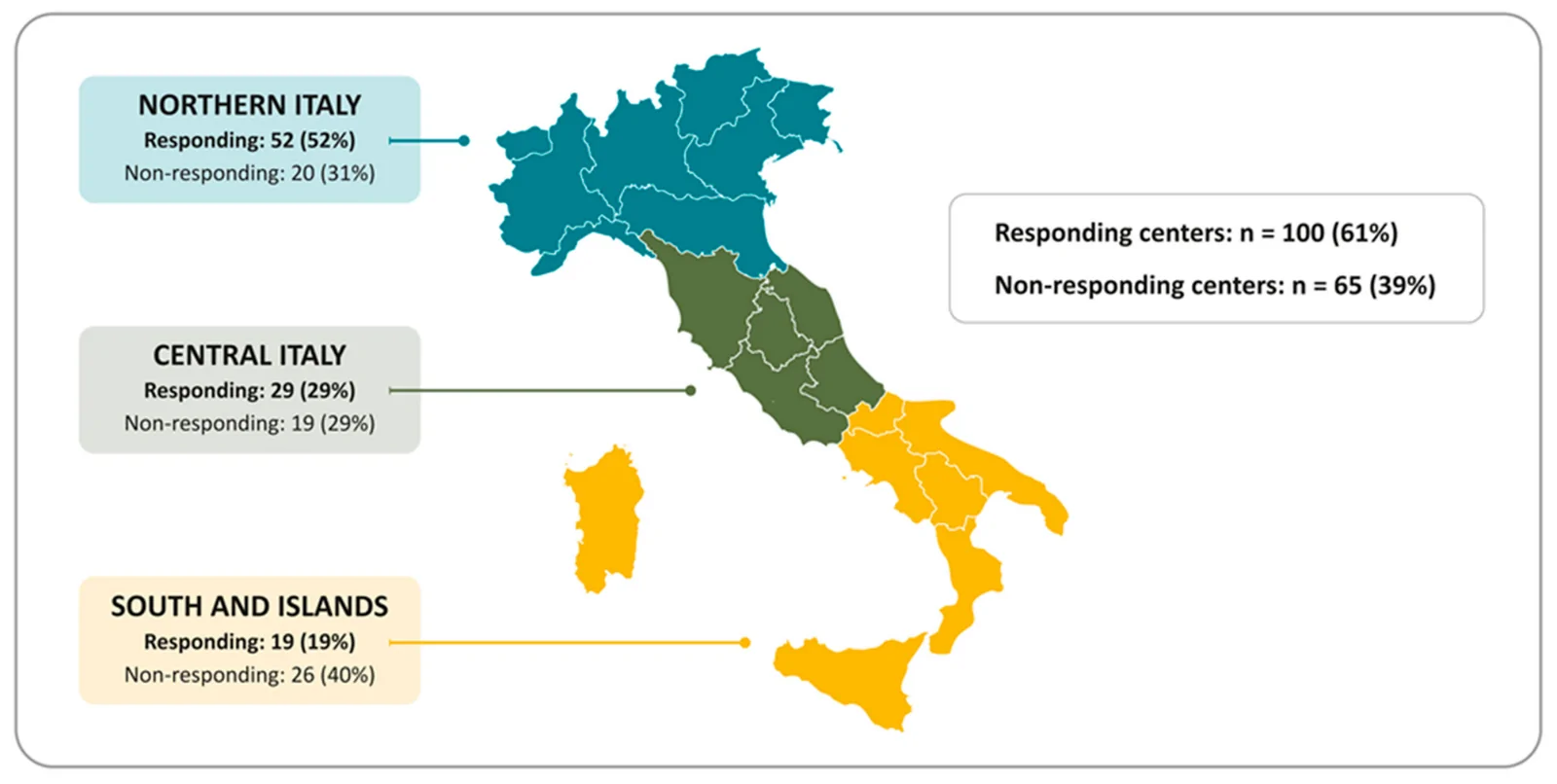
Geographical distribution of responding and non-responding oncology centers across the three Italian macro-regions.

A total of 100 of the 165 invited centers (61%) completed the survey. Figure 1 shows the geographical distribution of responding and non-responding centers across the three Italian macro-regions, with a higher number of participating centers from Northern Italy than from Central or Southern Italy and the Islands.

Overall, 51% of centers offer structured, consistently accessible regional nutrition programs, while 23% have hospital-based programs. Dedicated professionals responsible for nutritional screening are present in 86% of centers; these include nutritionists or dietitians (84%), clinical nutrition physician (44%), and dedicated nurses or oncologists (17%). Nutritional screening results are documented in patients’ medical records in 65% of centers.

Regarding nutritional assessment, 67% of centers perform it at the time of diagnosis; in 48% of cases, the assessment is carried out by oncologists; 50% by nutritionists or dietitians; and 30% by oncological nurses. This assessment is conducted for all patients in 46% of cases, while in 42% of cases, it is reserved for patients who are underweight or have reported weight loss.

Regarding the tools used, 69% of centers adopt tests validated by the ESPEN guidelines; specifically, 59% use the Malnutrition Universal Screening Tool (MUST). In terms of parameters assessed, 44% of centers base their assessments on weight and height, 25% also measure circumferences, 21% use bioimpedance analysis, and 10% use skinfold calipers. Furthermore, 84% of centers reassess nutritional status outside of the initial oncology visit, but only 15% do so systematically during follow-up.

At the same time, 59% of centers assess all patients’ dietary habits as part of their medical history, while 70% also consider physical activity. Nutrition recommendations are provided in 92% of centers, and in 54% of cases, to all patients. Furthermore, 59% provide informational materials on proper nutrition, of which 58% consist of printed brochures.

Regarding centers’ awareness of the importance of nutrition, 98% of centers believe it is important to provide informational materials during the first visit. Additionally, 97% consider outpatient clinical nutrition management, including nutritional monitoring and personalized prescriptions, to be useful. Finally, 99% of centers emphasize the importance of physical activity programs.

## 4. Discussion

Cancer patients often experience nutritional impairment due to treatment-related toxicities, metabolic alterations, surgery, or the cancer itself, making nutritional care an essential component of comprehensive oncology management. These factors may contribute to cancer-related malnutrition. This survey evaluated the integration of nutritional care into Italian oncology pathways, with particular attention to nutritional assessment at diagnosis and the availability of dedicated care pathways. The findings reveal a substantial gap between guideline recommendations and routine clinical practice and confirm observations from previous Italian and international studies [20,21,22,23]. Similar challenges have been reported in several countries, suggesting that difficulties in implementing nutritional care extend beyond the Italian context and reflect broader barriers to integrating supportive care into routine oncology practice [24]. Although ESPEN and AIOM guidelines recommend systematic nutritional screening for all cancer patients at diagnosis, our survey indicates that this recommendation has not yet been fully implemented [16,19]. Nutritional assessment at diagnosis was routinely performed in only 67% of centers. Among these, only 41% assessed all patients regardless of nutritional status, whereas the remaining centers limited assessment to selected high-risk groups. Consequently, malnutrition may remain underrecognized despite its well-established impact on treatment tolerance, quality of life, and survival [18,22,23].

Multiple factors may contribute to this implementation gap, including organizational constraints, limited consultation time, lack of standardized screening procedures, insufficient referral pathways to clinical nutrition services, and the tendency to prioritize anticancer treatment over supportive care interventions [25,26].

The marked heterogeneity observed across centers further reflects differences in local organization, institutional priorities, and resource allocation, resulting in unequal access to nutritional assessment and interventions. Consistent with this finding, the involvement of oncologists and oncology nurses in nutritional management suggests that multidisciplinary integration remains incomplete in many settings, despite ESPEN and AIOM recommendations that identify multidisciplinary nutritional care as a core component of high-quality oncology practice [16,19].

Important shortcomings also emerged in the nutritional assessment process. Although 84% of participating centers reported reassessing nutritional status beyond the initial oncology visit, only 15% performed systematic reassessment during follow-up. This is particularly relevant because nutritional status is dynamic and may deteriorate during treatment owing to therapy-related toxicities, disease progression, or declining functional status. Likewise, the limited use of validated screening tools and the inconsistent collection of dietary information may hinder early identification of patients at nutritional risk and delay timely, evidence-based interventions, indicating that current practice remains only partially aligned with ESPEN recommendations for repeated nutritional screening throughout the continuum of cancer care [16,26].

Current guidelines also recommend that nutritional counseling and patient education should be routinely integrated into oncology care. However, only about half of the participating centers systematically provide nutritional education to all patients, despite evidence supporting its role in improving treatment adherence, quality of life, and clinical outcomes [16,27].

Although healthcare professionals demonstrated a high level of awareness regarding the importance of nutrition, this did not consistently translate into routine clinical practice. Such a discrepancy, which is well-recognized in implementation science, suggests that knowledge alone is insufficient without adequate organizational support, resources, and standardized clinical pathways [28].

A similar gap emerged for physical activity. While almost all participating centers (99%) recognized the importance of exercise for cancer patients, only 70% routinely assessed patients’ physical activity during the initial evaluation. This discrepancy may reflect the absence of standardized assessment procedures, limited consultation time, and the perception that exercise counseling should be delegated to other healthcare professionals rather than incorporated into routine oncological assessment. Consequently, opportunities to identify physically inactive patients and provide tailored exercise recommendations or refer them to supportive care programs may be missed. Given the growing evidence supporting exercise as an integral component of cancer care, systematic assessment of physical activity should be incorporated into routine clinical practice alongside nutritional screening.

These findings further support the integration of nutrition specialists within multidisciplinary oncology teams to promote the early identification and management of nutritional risk.

Beyond clinical benefit, early nutritional screening and intervention may also reduce healthcare resource utilization and represent a cost-effective strategy by decreasing complications, intensive care utilization, and overall care costs [29,30,31,32].

From an organizational perspective, the adoption of measurable quality indicators, including screening rates, documentation of nutritional status, referral times to nutrition services, and monitoring of nutritional outcomes, could facilitate implementation and identify areas for improvement. Such measures may help healthcare systems reduce variability in clinical practice and promote a preventive, rather than reactive, model of nutritional care.

In this context, emerging AI-based tools may further support routine practice by enabling automated body composition assessment from oncological imaging, improving nutritional risk stratification, facilitating remote patient evaluation, and contributing to more personalized nutritional interventions [33].

The broad consensus among respondents regarding the need for educational initiatives, dedicated nutritional pathways, and structured care models indicates favorable conditions for improving the integration of nutritional care into routine oncology practice. Standardizing nutritional screening, strengthening multidisciplinary collaboration, and ensuring equitable access to nutritional services should represent key organizational priorities for reducing the current gap between guideline recommendations and clinical practice.

From a practical perspective, systematic nutritional screening should be implemented for all patients at the first oncology visit, using validated tools such as MUST or MST, with results routinely documented in the medical record [16]. In addition, body composition assessment should be more widely incorporated into clinical practice, including accessible techniques such as bioimpedance analysis (BIA), given its growing prognostic relevance in oncology and its potential contribution to individualized risk stratification [34,35,36]. Standardized educational materials should also be routinely made available to patients and include information on the role of physical activity in preventing and managing sarcopenia and cachexia, consistent with the growing evidence supporting combined nutritional and exercise interventions [27,37,38].

Future research should extend the present organizational perspective by incorporating patient-reported outcomes and patient experiences regarding access to, quality of, and satisfaction with nutritional care. In addition, qualitative or mixed-methods studies are needed to better understand the organizational, structural, and cultural barriers that limit the implementation of nutritional care, while prospective clinical studies should evaluate whether improved adherence to recommended organizational pathways translates into better nutritional status, treatment tolerance, quality of life, and survival. Future initiatives should also promote the integration of nutritional care into clinical trials and ensure equitable nationwide access to nutritional services, including the formal recognition of dietary consultations within the Essential Levels of Assistance (LEA), thereby reducing regional disparities in supportive cancer care.

### Limitations

This study has several limitations that should be acknowledged. First, although the survey achieved an acceptable response rate (61%), the geographic distribution of participating centers was not homogeneous, with greater representation from Northern Italy and lower participation from Southern Italy and the Islands. In addition, non-responding centers were proportionally more represented in Southern Italy and the Islands, which may have affected the representativeness of the sample. Because detailed information on the organizational characteristics of non-responding centers was unavailable, a formal non-response bias analysis could not be conducted. Therefore, the findings may not fully reflect nutritional care practices across all Italian oncology centers and should be interpreted with appropriate caution when generalizing the findings at the national level.

Second, although the questionnaire was developed by the CIPOMO working group based on current scientific recommendations and expert clinical experience, it did not undergo formal validation, including pilot testing or assessment of psychometric properties, before administration. In addition, because the survey relied on self-reported responses, some answers, particularly those regarding the perceived importance of nutrition and physical activity, may have been influenced by social desirability bias. Consequently, the very high endorsement rates observed for these items may reflect respondents’ awareness of current guideline recommendations and perceived professional expectations, rather than the actual and consistent implementation of these practices in routine clinical care.

Furthermore, some organizational terms used in the questionnaire, such as “structured nutritional care pathway” and “dedicated specialists”, were not accompanied by formal operational definitions and may therefore have been interpreted differently across participating centers. In addition, some response options regarding the timing of nutritional assessment may have been perceived as partially overlapping, potentially leading to heterogeneous interpretations among respondents. Future surveys should adopt more clearly defined and mutually exclusive response categories to improve clarity and facilitate data interpretation.

Finally, the survey was designed as an exploratory descriptive assessment of current clinical practice and evaluated organizational processes of nutritional care rather than patient-level clinical outcomes. Therefore, the present findings should not be interpreted as demonstrating improvements in nutritional status, treatment tolerance, quality of life, or survival. Consistent with the study’s descriptive design, no inferential statistical analyses were performed. Future studies using formally validated survey instruments and prospective comparative designs are warranted to further strengthen the evidence. Nevertheless, despite these limitations, the survey provides a valuable nationwide overview of current organizational practices and highlights important areas for improvement in the integration of nutritional care into oncology pathways.

## 5. Conclusions and Future Perspectives

The CIPOMO national survey provides a comprehensive overview of the current organization of nutritional care in Italian oncology centers, highlighting substantial variability in clinical practice and incomplete implementation of recommended nutritional pathways. Although awareness of the importance of nutritional care is widespread, systematic screening, timely assessment, and structured multidisciplinary management have not yet been consistently integrated into routine oncology practice. These findings underscore the need to standardize organizational pathways and strengthen the integration of nutritional care as an essential component of comprehensive cancer management, thereby promoting equitable access to evidence-based nutritional support for all patients from the time of diagnosis.

The present survey provides a valuable baseline for future national initiatives aimed at monitoring and improving nutritional care in oncology. Looking ahead, greater integration between oncology departments and community healthcare services may facilitate earlier and more accessible delivery of nutritional counseling and structured physical activity interventions throughout the cancer care pathway.

## Figures and Tables

**Table 1:** CIPOMO questionnaire.

No.	Question	Response Option	n/N (%)
**1**	Is there a regional nutritional care pathway for all oncology patients in your region?	Yes	51/100 (51%)
		No	26/100 (26%)
		It exists at the level of my hospital but not at the regional level	23/100 (23%)
**2**	Is nutritional screening documented in the electronic medical record or included in the patient’s health and social care documentation for patients receiving integrated home care?	Yes	65/100 (65%)
		No	35/100 (35%)
**3**	Does your center have healthcare professionals dedicated to the nutritional care of oncology patients?	Yes	86/100 (86%)
		No	14/100 (14%)
		If yes Which healthcare professionals are available for nutritional assessment of oncology patients? ^a^	
		- Dedicated nurse	15/86 (17%)
		- Dedicated oncologist	15/86 (17%)
		- Nutritionist/Dietitian	72/86 (84%)
		- Clinical nutrition physician	38/86 (44%)
**4**	Is nutritional assessment routinely performed at diagnosis (during or immediately after the first visit)?	Yes	67/100 (67%)
		No	33/100 (33%)
		If yes (A) ^a^ Who performs the initial nutritional assessment?	
		- Nurse	20/67 (30%)
		- Oncologist	32/67 (48%)
		- Nutritionist/Dietician	33/67 (49%)
		- Clinical nutrition physician	9/67 (13%)
		- Corporate nutrition team	7/67 (10%)
		If yes (B) ^a^ Nutritional assessment is performed in:	
		- All patients, regardless of body weight	31/67 (46%)
		- Only underweight patients or those with reported weight loss	28/67 (42%)
		- Underweight patients or those with reported weight loss, and overweight patients	15/67 (22%)
		- Only patients who request it	2/67 (3%)
		If yes (C) ^a^ Nutritional assessment is performed in:	
		- All patients regardless of cancer type, disease stage, or care setting	26/67 (39%)
		- All patients eligible for active treatment, regardless of cancer site	23/67 (34%)
		- Only patients with specific cancer sites (e.g., GI tract, head and neck), regardless of therapeutic indication	23/67 (34%)
		- Only hospitalized patients	6/67 (9%)
		- Other *	9/67 (13%)
**5**	Does your center use an ESPEN guideline-validated nutritional screening tool to assess weight loss and the possible presence of protein-energy malnutrition?	Yes	69/100 (69%)
		No	31/100 (31%)
		If yes ^a^ Which nutritional screening tools are used?	
		- MUST (Malnutrition Universal Screening Tool)	41/69 (59%)
		- MST (Malnutrition Screening Tool)	8/69 (12%)
		- MNA-SF (Mini Nutritional Assessment Short-Form)	11/69 (16%)
		- NRS 2002 (Nutritional Risk Screening 2002)	12/69 (17%)
		If No Do you use any other nutritional screening tool?	
		- Yes (please specify) **	8/31 (26%)
		- No	24/31 (77%)
**6**	Which parameters are measured to assess the nutritional status of oncology patients in your center? ^a^	Weight and height	60/100 (60%)
		Weight, height, and anthropometric circumference measurements	34/100 (34%)
		Skinfold thickness (skinfold caliper)	13/100 (13%)
		Bioelectrical Impedance Analysis (BIA)	28/100 (28%)
**7**	During medical and/or nursing history taking, are patients asked about their dietary habits even in the absence of weight loss?	Yes	59/100 (59%)
		No	41/100 (41%)
		If yes (A) Are patients asked whether they follow any specific dietary regimens?	
		- Vegetarian diet	11/59 (19%)
		- Vegan diet	5/59 (8%)
		- Ketogenic diet	4/59 (7%)
		- Intermittent fasting	1/59 (2%)
		- Other ***	34/59 (58%)
		If yes (B) ^a^ Are patients asked whether they are being followed by a nutrition professional?	
		- Yes	50/59 (85%)
		- No	9/59 (15%)
**8**	At your center, are oncology patients provided with information on?	Yes	92/100 (92%)
		No	8/100 (8%)
		If yes (A) Which patients receive information on nutrition?	
		- All patients, regardless of cancer type, disease stage, or need for active treatment	49/92 (53%)
		- Only patients with an indication for active treatment	17/92 (18%)
		- Only patients who request it	9/92 (8%)
		- Only patients with conditions that may interfere with food intake and absorption (e.g., gastrointestinal, hepatobiliary-pancreatic, or head and neck cancers)	16/92 (17%)
		If yes (B) ^a^ Which healthcare professional provides information about proper nutrition?	
		- Dedicated nurse	12/89 (13%)
		- Dedicated oncologist	28/89 (30%)
		- Dedicated nutritionist/dietitian	47/89 (51%)
		- Clinical nutrition physician	18/89 (20%)
		- Other ^§^	15/89 (16%)
**9**	Is nutritional status assessed at your center outside of the first oncology visit?	Yes	84/100 (84%)
		No	16/100 (16%)
		If yes, when? (A)	
		- At one of the visits prior to therapy	22/84 (26%)
		- At follow-up visits, for all patients	8/84 (10%)
		- At follow-up visits, only in certain patient groups (stage and/or cancer type and/or previous CT treatment)	24/84 (29%)
		- At visits following the first visit, systematically	14/84 (17%)
		- At visits following the first visit, sporadically	26/84 (31%)
**10**	Does your center provide patients with educational material on the importance of proper nutrition?	Yes	59/100 (59%)
		No	41/100 (41%)
		If yes What type of educational material is provided?	
		- Brochure provided by the oncology network	15/59 (25%)
		- Brochure prepared by the center’s healthcare professionals	34/59 (58%)
		- References to websites providing educational materials (e.g., Ministry of Health website, websites of relevant institutions, or patient association websites)	14/59 (24%)
		- Other ^#^	2/59 (3%)
**11**	Do you consider it would be useful to establish nutritional education groups for patients with similar clinical characteristics (based on disease type and site, as well as type of surgery and/or medical or physical therapy received)?	Yes	93/100 (93%)
		No	7/100 (7%)
		If yes ^a^ Which approach do you think would be most appropriate for managing these groups?	
		- In-person at each treatment center	54/93 (58%)
		- Online at the local or regional level	19/93 (20%)
		- Online at the national level, with the involvement of patient associations that already organize similar initiatives	9/93 (10%)
		- Online at the national level, with the involvement of patient associations and scientific societies (e.g., AIOM) that already organize similar initiatives	34/93 (37%)
**12**	Do you think it would be useful to develop an informational brochure on the importance of nutrition in oncology patients to be provided during the first visit?	Yes	98/100 (98%)
		No	2/100 (2%)
**13**	Do you consider outpatient clinical nutrition management, including nutritional status monitoring and personalized dietary prescriptions, to be useful?	Yes	97/100 (97%)
		No	3/100 (3%)
		If yes Which patients should be offered this program?	
		- All patients, with an individualized care pathway based on the nutritional risk identified through screening	10/97 (10%)
		- Only underweight patients	2/97 (2%)
		- Underweight and severely overweight patients	3/97 (3%)
		- Only patients undergoing systemic treatments that may affect their ability to eat	81/97 (86%)
**14**	Physical activity (exercise) helps prevent sarcopenia, depression, immune deficiency in oncology patients, and cachexia and should therefore be encouraged. During the initial assessment, are oncology patients in your center asked about their level of physical activity?	Yes	70/100 (70%)
		No	30/100 (30%)
**15**	Do you think an educational initiative on this topic would be useful for all oncology patients?	Yes	99/100 (99%)
		No	1/100 (1%)

^a^ Multiple answers allowed. * Other (individual responses): patients considered at nutritional risk; patients attending the first oncology visit; day-hospital patients; candidates for gastrointestinal surgery (pre- and post-operative); patients with weight loss or anorexia; underweight or overweight patients; patients with head and neck or gastrointestinal cancers; older adults and patients receiving palliative care; patients referred by the oncologist to the nutritionist. ** Yes, please specify (individual responses): non-formalized nutritional screening; PG-SGA used in combination with MNA-SF as part of a future project; institution-specific screening tool; PG-SGA and MUST; MST according to the Regional Oncology Network recommendations; patient-completed questionnaire; referral to the nutrition service; protein assessment and adherence to the Mediterranean diet. *** Other (similar responses grouped): type of diet/dietary regimen (*n* = 11); dietary habits (*n* = 6); all of the above (*n* = 6); personalized nutritional plans (*n* = 1); dietary preferences and religious restrictions (*n* = 1); use of dietary supplements/meal replacements (*n* = 1). Three responses did not provide an additional specification (“No”, “I answered no”, or equivalent). ^§^ Other (similar responses grouped): medical oncologist (*n* = 6); disease-/area-specific medical oncologist (*n* = 2); nutritionist (*n* = 1); non-dedicated nurse (*n* = 1); medical oncologist and non-dedicated nurse (*n* = 1); multidisciplinary healthcare team (*n* = 2); according to the patient’s disease and nutritional status (*n* = 1); professionals involved in the care of oncology patients rather than dedicated staff (*n* = 1). ^#^ Other (individual responses): dietitian’s recommendations; AIMaC educational booklets; meetings organized by local volunteer associations; FAVO Caregiver Academy. **Note:** Percentages were calculated using the number of respondents to each question as the denominator (N). The denominator is reported for each response category. Abbreviations: AIOM, Associazione Italiana di Oncologia Medica (Italian Association of Medical Oncology); CT, Chemotherapy; BIA, Bioelectrical Impedance Analysis; ESPEN, European Society of Clinical Nutrition and Metabolism; MNA-SF, Mini Nutritional Assessment Short-Form; MUST, Malnutrition Universal Screening Tool; NRS 2002, Nutritional Risk Screening 2002; MST, Malnutrition Screening Tool.

## Data Availability

The survey data supporting this manuscript are available from the corresponding author upon reasonable request.
